# Leptin levels after subarachnoid haemorrhage are gender dependent

**DOI:** 10.1186/s40064-016-2321-3

**Published:** 2016-05-27

**Authors:** Cecilia Lindgren, Silvana Naredi, Stefan Söderberg, Lars-Owe Koskinen, Magnus Hultin

**Affiliations:** Department of Surgical and Perioperative Sciences, Anaesthesiology and Intensive Care, Umeå University, Umeå, Sweden; Department of Anaesthesiology and Intensive Care, Institute of Clinical Sciences, Sahlgrenska Academy at the University of Gothenburg, Gothenburg, Sweden; Department of Public Health and Clinical Medicine, Medicine and Heart Centre, Umeå University, Umeå, Sweden; Department of Pharmacology and Clinical Neuroscience, Neurosurgery, Umeå University, Umeå, Sweden

**Keywords:** Leptin, Gender, Subarachnoid haemorrhage, Organ failure, Outcome

## Abstract

**Background:**

Subarachnoid hemorrhage (SAH) is a neurological disease where the majority of the patients are critically ill. The adipokine leptin has in cerebral emergencies been related to severity of disease and to adverse outcome. The aim of this study was to examine leptin levels over time after SAH and associations to gender, age, body mass index, severity of disease, parenteral lipids, systemic organ failure and outcome.

**Methods:**

Prospective observational study in 56 patients. Leptin was obtained 0–240 h after SAH, in 48 h intervals. Severity of disease was assessed with the Hunt and Hess score, organ failure with the sequential organ failure assessment score, and outcome with Glasgow outcome scale. Leptin levels in the SAH group were compared with controls from the same geographical area.

**Results:**

At admission, Leptin was significantly higher in SAH patients compared to controls, both in female (28.6 ± 25.6 vs 13.0 ± 2.3 ng/mL, p = 0.001) and male patients (13.3 ± 8.4 vs 4.3 ± 0.7 ng/mL, p = 0.001). Leptin levels remained stable over time. Female patients had significantly higher leptin levels than male patients, and deceased female patients had higher leptin levels than female survivors (85.5 ± 20.5 vs 50.5 ± 34.6, n = 4/35, p < 0.05). Leptin levels did not differ between male survivors and non-survivors. Leptin levels were not associated with severity of disease, organ failure or parenteral lipids.

**Conclusion:**

Leptin levels were significantly higher in both male and female patients compared to controls. Higher leptin levels were related to outcome and organ failure in women but not in men. When analysing leptin levels gender-related differences should be considered.

## Background

Subarachnoid haemorrhage (SAH) caused by the rupture of a cerebral aneurysm is a serious form of stroke, where a majority of the patients are initially critically ill (Diringer et al. 2011). The SAH produces an initial global ischemic brain injury that stimulates the sympathetic nervous system and starts an inflammatory process (Zetterling et al. 2010; Naredi et al. 2000).

This inflammatory process not only affects the brain but also gives rise to a systemic inflammatory response syndrome (SIRS), with release of inflammatory mediators such as cytokines and adipokines from adipocytes (Harle and Straub 2006).

In critical illness, the adipose tissue has gone from being just storage of energy to become an active organ taking part in the inflammatory process (Marques and Langouche 2013).

The adipokine leptin is a 16 kDa large protein that is mainly produced by the white adipose tissue and regulates energy balance (Zhang et al. 1994). Food deprivation and fasting decrease circulating leptin levels whereas food intake increases leptin levels (Gosmanov et al. 2010; Garcia-Lorda et al. 2003; Evans et al. 2001). Leptin also acts as a cytokine and is as such a regulator of innate immunity (Zhang et al. 1994; Schwartz et al. 2000). Leptin levels are higher in women than in men, and circulating levels strongly associates with fat mass, measured for example as body mass index (BMI). Leptin has a cyclic secretion and normally peaks at night, but the circadian rhythm has been reported as lost during critical illness (Bornstein et al. 1998; Simon et al. 1998). Studies in critically ill patients have shown variations in leptin levels and both normal and elevated leptin levels have been reported to correlate to inflammatory markers, body temperature and cortisol levels (Bornstein et al. 1998; Papathanassoglou et al. 2001; Koch et al. 2010; Chen et al. 2014; Grigoras et al. 2014; Yousef et al. 2010).

Leptin-receptors have been located to several parts of the central nervous system (CNS), most abundant in hypothalamus but also in the brainstem (Schwartz et al. 2000; Grill and Hayes 2012). Increased leptin levels have been reported in patients with intracerebral haematomas (ICH), SAH and ischemic stroke. Further, elevated leptin levels have been shown to predict both ischemic and haemorrhagic stroke (Soderberg et al. 2004; Kim et al. 2012; Kantorova et al. 2011; Dong et al. 2010; Du et al. 2013; Fan et al. 2013; Huang et al. 2014; Zhang et al. 2007; Zhao et al. 2012).

The aim of this study was to examine if leptin levels change over time in the acute phase of SAH and if the leptin levels were associated with gender, age, BMI, severity of disease, administration of parenteral lipids, systemic organ failure and outcome.

## Patients and methods

This is a prospective observational study of patients with SAH due to a ruptured cerebral aneurysm. The patients were treated at the department of neurosurgery, Umeå University hospital (UUH), Sweden and were consecutively included from March 2008 until September 2009. The department of neurosurgery provides neurosurgical care to the four northernmost counties in Sweden with a total of 878,000 inhabitants (in 2008).

Inclusion criteria were; SAH caused by a cerebral aneurysm, verified by digital subtraction angiography (DSA) or CT angiography (CTA), age ≥ 18 years, and arrival at UUH ≤ 48 h after the first symptom of SAH that brought the patient to hospital. Exclusion criteria were; pregnant/lactating woman, earlier SAH or intracranial surgery.

At admission to UUH, a medical history was obtained regarding co-morbidities and medication. The length and weight of the patient was noted and BMI was calculated. BMI >25 was considered as overweight.

The severity of the SAH was assessed with the Hunt–Hess (H&H) score. H&H extends from 1; minimal symptoms, to 5; deep coma. H&H 3–5 is considered as a severe clinical condition and H&H 1–2 as a less severe clinical condition (Hunt and Hess 1968). The H&H scores were obtained from the first clinical examination made by a physician.

Sequential Organ Failure Assessment (SOFA) score was used for assessment of organ failure. SOFA extends from zero; no organ failure, to four; pronounced organ failure. SOFA is based on observations during a 24-h interval and the most abnormal value from the preceding 24 h is used. Six different organs are evaluated; respiration, hematology, hepatic, cardiovascular, renal and central nervous system (CNS) (Vincent et al. 1996). SOFA CNS was not used in this study, since a majority of the patients were sedated at some time during the study period. A daily total SOFA score (SOFA_sum_) was calculated including all organ systems except CNS. In order to find out whether severe organ failure was related to leptin levels, patients were divided in two groups;

1. Severe organ failure; SOFA ≥ 3 in at least one organ system at any time during the study period.
2. No severe organ failure; SOFA < 3 at all times, in all organ systems, during the study period.

### Leptin

The first sample for leptin analysis was obtained at admission within 0–48 h from the first symptoms of SAH. Consequent samples were thereafter obtained in 48 h intervals i.e. in the time intervals: 49–96, 97–144, 145–192, 193–240 h after the SAH. Blood sampling was performed between 8 and 11 AM. Leptin levels at admission were compared to levels in a control population from the same area in northern Sweden. This control group emerged from the Northern Sweden MONICA survey (MONItoring of trends and determinants in Cardiovascular disease) (Eriksson et al. 2011).

A double-antibody radioimmunoassay from Millipore Linco, St. Charles, MO, USA, was used for both SAH patients and the control population, and the total coefficient of variation for leptin was 4.7 % at both low (2–4 ng/mL) and high (10–15 ng/mL) levels (Lilja et al. 2010).

### Treatment protocol

A local treatment protocol for SAH was used, coherent in its key parts with strategies given by the Neurocritical Care Society’s Multidisciplinary Consensus Conference and American Heart Association (AHA) guidelines (Diringer et al. 2011; Connolly et al. 2012). The protocol includes normoventilation, normovolemia and keeping sodium, glucose, haemoglobin and albumin within normal limits. The intention was to secure the cerebral aneurysms within 24 h after arrival, either by surgical or endovascular treatment. Early enteral nutrition was encouraged and was started as early as within the first 48 h after admission. Parenteral nutrition was administered using StructoKabiven^®^, a mix of glucose, amino acids and lipids (Fresenius Kabi 2015). The lipids in StructoKabiven^®^ is based on StructoLipid^®^ emulsion containing an interesterified mixture of equimolar amounts of long chain triglycerides (LCT) from soybean oil and medium chain triglycerides synthetically derived from a mixture of coconut and/or palm kernel oil (MCT). The equimolar relation between MCT and LCT translates to 34 % (V/V) and 66 % (v/v), respectively (Fresenius Kabi, Uppsala, Sweden).

General anaesthesia was performed with thiopental, sevoflurane and remifentanil. In ventilated patients at the intensive care unit the most commonly used sedative agent was propofol. Propofol is an intravenous anaesthetic agent dissolved in Intralipid^®^ (McKeage and Perry 2003). The propofol preparation from the manufacturers contains, in addition to 10 or 20 mg/mL propofol, soybean-oil (100 mg/mL), egg yolk phospholipids (12 mg/mL), glycerol (22.5 mg/mL), and sodium edetate (0.055 mg/mL). In case of prolonged controlled ventilation due to neurological or respiratory needs a change from propofol to midazolam was usually made. Thiopental was added in patients with intracranial hypertension, Fentanyl was given in continuous infusion for analgesia. All patients were treated with intravenous nimodipine (0.2 mg/mL dissolved in ethanol (96 %) with additives of macrogol 4000, sodium citrate and citric acid (Nimotop^®^, Bayer). The dose administered aimed at 10 mL/h. Patients in need for prolonged sedation and ventilation had continuous intracranial pressure monitoring with an external ventricular drain or an intraparenchymal sensor (Codman^®^ MicroSensor™, Codman & Shurtleff Inc. Raynham, MA, USA).

At a follow-up visit performed approximately 1 year after the SAH, an independent research nurse scored the patients according to Glasgow outcome scale (GOS) by structured interviews (Jennett and Bond 1975). GOS extends from 1; dead to 5; good recovery. Favorable outcome was defined as GOS 4–5 and unfavorable outcome as GOS 1–3.

### Statistics

The statistical software package, Prism, version 5.0 (GraphPad Software Inc., CA, USA) was used for statistical analyses. Data are presented as mean ± SD, median (range) or percentage. The non-parametric Mann–Whitney test was used for comparison of leptin and different clinical parameters. Wilcoxon-signed-rank-test was used for comparison of leptin levels at admission with different time points and before/after administration of lipids. Fisher’s exact test was used for differences in SOFA score between female and male patients. Spearman’s test was used for correlation of leptin levels and and SOFA score. Statistical significance was set at p < 0.05.

## Results

Fifty-six patients were included in the study, 39 female (70 %) and 17 male (30 %) patients, and median ages were 59 (31–82) years in female and 63 (26–77) years in male patients.

**Fig. 1:**
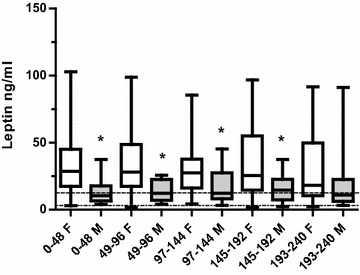
Leptin (ng/mL) in female and male patients at different time intervals, 0–240 h after subarachnoid haemorrhage (SAH) *F* female patients (*white bars*), *M* male patients (*grey bars*). *Significant difference *p* < 0.05. Leptin values at admission, 0–48 h, after SAH and thereafter distributed into 48 h intervals: 49–96, 97–144, 145–192, 193–240 h after SAH. Male patients: 0–48 h: n = 17, 49–96 h: n = 17, 97–144 h: n = 16, 145–192 h: n = 16, 193–240 h: n = 16. Female patients: 0–48 h: n = 37, 49–96 h: n = 37, 97–144 h: n = 35, 145–192 h: n = 34, 193–240 h: n = 34. *Dotted lines* normal values for males: 4.3 ± 0.7 ng/mL/females: 13.0 ± 2.3 ng/mL

In total, 259 samples of leptin were obtained within the first 240 h after the SAH, in average 5 (2–5) samples per patient. Leptin levels did not change significantly compared to admission (0–48 h) over time (49–240 h) after the SAH, neither in male nor in female patients. Male patients had significantly lower leptin levels compared to female patients 0–192 h after SAH (Fig. 1).

Leptin at admission was significantly higher in SAH patients, compared to controls from the Northern Sweden MONICA health study, both in female (28.6 ± 25.6 vs 13.0 ± 2.3 ng/mL, p = 0.001) and in male patients (13.3 ± 8.4 vs 4.3 ± 0.7 ng/mL, p = 0.001).

Leptin values at admission in relation to different clinical parameters are given in Table 1. Peak and nadir leptin values in each patient 0–240 h after SAH in relation to different parameters are given in Table 2.

**Table 1:** Leptin (ng/mL) at admission (0–48 h) after SAH

Parameter	Number of samples	Leptin (ng/mL)	Sig.
Gender			
Female/male	37/17	35.5 ± 25.5/14.4 ± 7.8	<0.0001
Age^a^			
Female			
≤59/>59	18/19	23.8 ± 16.9/46.5 ± 27.6	ns
Male			
≤63/>63	8/9	15.6 ± 10.4/11.3 ± 6.1	ns
BMI^b^			
Female			
≤25/>25	20/17	27.7 ± 19.4/28.3 ± 2.9	<0.05
Male			
≤25/>25	11/6	24.5 ± 16.4/26.9 ± 1.6	ns
Hypertension^c^			
Female			
Yes/no	10/27	33.1 ± 18.0/36.3 ± 28.0	ns
Male			
Yes/no	8/9	17.8 ± 9.7/9.3 ± 4.6	<0.05
Hunt and Hess^d^			
Female			
1–2/3–5	17/20	38.9 ± 29.1/32.6 ± 22.2	ns
Male			
1–2/3–5	12/5	12.7 ± 9.2/14.8 ± 6.7	ns
GOS^e^			
Female			
1–3/4–5	8/29	46.7 ± 33.9/32.4 ± 22.4	ns
Male			
1–3/4–5	6/11	17.7 ± 11.6/10.9 ± 5.3	ns
Deceased^f^			
Female			
Yes/no	4/33	44.6 ± 33.5/31.9 ± 22.5	<0.05
Male			
Yes/no	3/14	16.2 ± 7.8/12.7 ± 8.7	ns
SOFA^g^			
Female			
0–2/3–4	11/26	39.0 ± 28.2/34.0 ± 24.7	ns
Male			
0–2/3–4	4/13	12.9 ± 5.2/13.4 ± 9.4	ns

In two female patients, blood samples from 0 to 48 h are not obtained

^a^Median age in female patients was 59 (31–82) and in male patients 63 (26–77) years

^b^ *BMI* body mass index; females patients median 24 (18–44), male patients median 25 (22–30)

^c^Patients with pharmacologically treated hypertension at admission

^d^ *H&H* Hunt and Hess score for classifying the severity of the SAH; 1 asymptomatic/minimal headache; 2 moderate/severe headache, no neurological deficit other than cranial nerve palsy; 3 drowsiness, confusion or mild focal deficit; 4 stupor, moderate to severe hemiparesis; 5 deep coma, moribund appearance. H&H 1–2 = less severe clinical condition, H&H = 3–5 severe clinical condition

^e^ *GOS* Glasgow outcome scale. (1) Dead, (2) Vegetative state, (3) Severe disability, (4) Moderate disability, (5) Good recovery. GOS 1–3 = Unfavourable outcome, GOS 4–5 = favourable outcome

^f^Seven patients died during the study period, three males and four females. Median time to death was 20 (9–39) days

^g^Sequential Organ Failure Assessment (SOFA) scores organ failure from zero; no organ failure, to four; pronounced organ failure. Six different organ systems are scored; respiration, haematology, hepatic, cardiovascular, renal and central nervous system (CNS). The worst value obtained during a 24-h period is used. In this study the SOFA CNS score was not used, Patients were divided in; severe organ failure ≥3 and not severe organ failure 0–2 at any time during the period studied

**Table 2:** Peak and mean Nadir values of leptin (ng/mL) 0–240 h after SAH

Parameter	Number of samples	Peak leptin (ng/mL)	Sig.	Nadir leptin (ng/mL)	Sig.
Gender					
F/M	(39/17)	54.1 ± 35.0/28.9 ± 23.7	ns	21.7 ± 16.0/9.3 ± 6.3	<0.001
Age^a^					
Female					
≤59/>59	(18/21)	53.6 ± 42.0/54.4 ± 28.6	ns	18.8 ± 14.2/24.2 ± 17.3	ns
Male					
≤63/>63	(10/7)	36.3 ± 28.5/18.3 ± 7.8	ns	10.8 ± 7.4/7.1 ± 3.5	ns
BMI^b^					
Female					
≤25/>25	(23/16	41.3 ± 27.8/72.3 ± 36.8	<0.05	14.5 ± 8.8/32.0 ± 18.4	<0.001
Male					
≤25/>25	(11/6)	25.0 ± 23.1/36.0 ± 25.3	ns	7.8 ± 5.6/12.1 ± 6.9	ns
Hypertension^c^					
Female					
Yes/no	(11/28)	65.3 ± 38.3/49.6 ± 33.3	ns	25.5 ± 15.9/20.2 ± 16.1	ns
Male					
Yes/no	(8/9)	40.7 ± 30.2/18.4 ± 7.8	ns	12.4 ± 7.6/6.5 ± 3.1	ns
H&H^d^					
Female					
1–2/3–4	(28/11)	51.0 ± 36.0/61.8 ± 32.5	ns	21.8 ± 17.5/21.4 ± 12.0	ns
Male					
1–2/3–4	(12/5)	27.4 ± 20.2/32.6 ± 33.1	ns	9.3 ± 6.0/9.3 ± 7.6	ns
GOS^e^					
Female					
1–3/4–5	(9/30)	56.6 ± 34.7/53.3 ± 35.6	ns	26.8 ± 21.9/20.1 ± 13.9	ns
Male					
1–3/4–5	(6/11)	34.4 ± 29.2/25.9 ± 21.1	ns	11.7 ± 8.8/8.0 ± 4.3	ns
Deceased^f^					
Female					
Yes/No	(4/35)	85.5 ± 20.5/50.5 ± 34.6	<0.05	38.1 ± 22.9/19.8 ± 14.3	<0.05
Male					
Yes/no	(3/14)	42.0 ± 42.7/26.1 ± 19.1	ns	12.1 ± 8.9/8.7 ± 5.8	ns
SOFA^g^					
Female					
0–2/3–4	(11/26)	49.1 ± 30.9/56.0 ± 36.8	ns	17.6 ± 9.7/23.3 ± 17.8	ns
Male					
0–2/3–4	(8/9)	18.0 ± 10.1/38.5 ± 28.3	ns	8.0 ± 7.0/10.5 ± 5.6	ns

Peak leptin value and nadir leptin value in each patient 0–240 h after SAH

*F* female patient, *M* male patient

^a^Median age in females was 59(31–82) years and in males 63(26–77) years

^b^ *BMI* Body mass index, females median 24 (18–44), males median 25 (22–30)

^c^Pharmacologically treated hypertension

^d^ *H&H* Hunt and Hess is scale for classifying the severity of the SAH; 1 asymptomatic/minimal headache; 2 moderate/severe headache, no neurological deficit other than cranial nerve palsy; 3 drowsiness, confusion or mild focal deficit; 4 stupor, moderate to severe hemiparesis; 5 deep coma, moribund appearance. H&H 1–2 = less severe clinical condition, H&H = 3–5 severe clinical condition

^e^GOS Glasgow outcome scale. (1) Dead, (2) Vegetative state, (3) Severe disability, (4) Moderate disability, (5) Good recovery. GOS 1–3 = Unfavourable outcome, GOS 4–5 = favourable outcome

^f^Seven patients died during the study period, three males and four females. Median time to death was 20 days (9–39)

^g^Sequential Organ Failure Assessment (SOFA) scores organ failure from zero; no organ failure, to four; pronounced organ failure. Six different organ systems are scored; respiration, haematology, hepatic, cardiovascular, renal and central nervous system (CNS). The worst value obtained during a 24-h period is used. In this study the SOFA CNS score was not used, Patients were divided in; severe organ failure ≥3 and no severe organ failure 0–2 at any time during the period studied

**Fig. 2:**
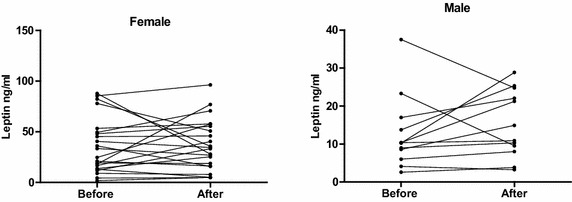
Leptin (ng/mL) before and 48 h after start of intravenous lipid infusion. Leptin in ng/mL. Female patients: Before; 36.0 ± 27.7 and after; 36.8 ± 25.1 start of intravenous lipid infusion. n = 22 pairs, non-significant. Male: Before: 12.7 ± 9.6 and after 15.2 ± 8.9 start of intravenous lipid infusion. n = 11 pairs, non-significant

In 22 female and in 12 male patients, leptin levels before and after start of intravenous lipid emulsion administration, as part of parenteral nutrition and/or sedation with propofol, were analysed. There was no significant difference between leptin levels before, and 48 h after start of intravenous lipid emulsion neither in female (36.0 ± 27.3 vs 36.8 ± 25.1 ng/mL) nor in male patients (12.7 ± 9.6/15.2 ± 8.8 ng/mL) (Fig. 2).

Patients *with* severe organ failure (SOFA ≥ 3) in at least one organ system at any time during the study period or patients *without* severe organ failure (SOFA score <3) in all organ systems during the study period is given in Table 3. The only difference found between female and male patients was a significantly higher incidence of circulatory failure in women at 49–144 h after SAH (Table 3).

**Table 3:** Patients with severe organ failure (SOFA ≥ 3) in different organ systems

Time interval	Respiration			Cardiovascular			Haematology	Hepatic	Renal
	Female n (%)	Male n (%)	Sig.	Female n (%)	Male n (%)	Sig.	Female/male n (%)	Female/male n (%)	Female/male n (%)
0–48	15/39 (38)	5/17 (29)	ns	22/39 (56)	11/17 (65)	ns	0	0	0
49–96	17/38 (45)	7/17 (41)	ns	10/38 (26)	0	p < 0.0001	0	0	0
97–144	15/38 (39)	7/16 (44)	ns	6/38 (16)	0	p < 0.0001	0	0	0
145–192	14/35 (40)	16/6 (38)	ns	1/35 (3)	0	ns	0	0	0
193–240	7/35 (20)	5/16 (31)	ns	1/35 (3)	0	ns	0	0	0

n = number of patients

ns = non-significant

s = significant difference p < 0.05

Leptin values in patients with a severe organ failure defined as SOFA ≥ 3 in at least one organ system at any time during the study period

Sequential Organ Failure Assessment (SOFA) scores organ failure from zero; no organ failure, to four; pronounced organ failure. Six different organ systems are scored; respiration, haematology, hepatic, cardiovascular, renal and central nervous system (CNS). The worst value obtained during a 24-h period is used. In this study the SOFA CNS score was not used

**Fig. 3:**
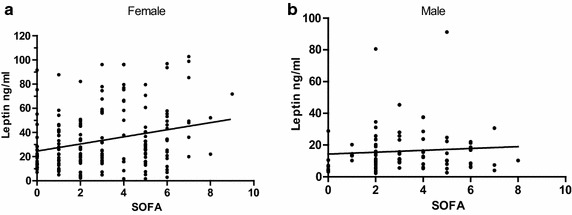
Correlation between SOFA_sum_ score and leptin levels. Sequential Organ Failure Assessment (SOFA) scores organ failure from zero; no organ failure, to four; pronounced organ failure. Six different organ systems are scored; respiration, haematology, hepatic, cardiovascular, renal and central nervous system (CNS). The worst value obtained during a 24-h period is used. In this study the SOFA CNS score was not used. Every days SOFA are summarized = SOFA_sum_. The SOFA sum was correlated to the leptin value taken within the same 24 h interval during the study period of 0–240 h. **a** Female patients: Spearman’s r = 0.25, CI (0.10–0.39), p = 0.0007, n = 177 pairs. **b** Male patients. No correlation, CI (−0.1 to 0.3), p = 0.3, n = 82 pairs

There was a correlation between SOFA_sum_ and leptin obtained during the same 24 h interval in female (Spearman’s r = 0.25, CI (0.10–0.39), p = 0.0007) but not in male patients (Fig. 3).

**Fig. 4:**
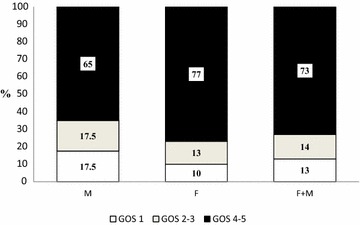
Outcome assessed with Glasgow Outcome scale. The follow-up was performed median 13 (6 ± 24) months after the SAH. GOS = Glasgow outcome scale. GOS 1–3 = Unfavourable outcome (GOS 1 = dead, GOS 2 = vegetative, GOS 3 = severe disability). GOS 4–5 = Favourable outcome (GOS 4 = moderate disability, GOS 5 = Good outcome)

Seven patients died during the study period (4 female and 3 male patients), median time to death was 20 (9–39) days. GOS 1–3 (unfavourable outcome) was found in 15/56 (27 %) patients and GOS 4–5 (favourable outcome) was found in 41/56 (73 %) patients, Fig. 4. Deceased female patients had both significantly higher leptin levels at admission and higher peak and nadir leptin levels during the study period than female survivors. In male patients, leptin levels did not differ between survivors and non-survivors (Tables 1, 2).

A follow-up visit was performed median 13 (6 ± 24) months after the SAH.

## Discussion

Compared to male patients, female SAH patients had significantly higher leptin values at admission and during the first 192 h after the SAH. This is not surprising since the levels of leptin are normally significantly higher in females compared to males, even when leptin is corrected for differences in BMI (Rosenbaum et al. 1996). This difference in gender has been observed already during childhood, and is actually starting already at birth (Wauters et al. 2000). When dividing the patients at median age, no difference related to age, could be found in SAH patients, although leptin levels are reported to decrease with age, independent of BMI, with a more pronounced decrease in women (Janeckova 2001). Leptin levels in SAH patients at admission were significantly elevated compared to controls, this is in accordance with other studies in critically ill patients and in patients with other acute cerebral diseases (Papathanassoglou et al. 2001; Grigoras et al. 2014; Yousef et al. 2010; Fan et al. 2013). Severe respiratory and cardiovascular organ failure, defined as SOFA score ≥3 was observed in up to 45 % of the patients. No severe haematological, hepatic or renal organ failure was detected during the study period. Using SOFA score ≥3 as a marker for severe organ failure, no significant difference in leptin levels could be found in patients with or without severe organ failure in this study. In female patients, a correlation between the leptin value and the SOFA sum score could be found, indicating a gender dependent reaction to leptin in critical illness.

There was no significant change in leptin levels over time (0–240 h) after SAH, neither in female nor in male patients. Both decreasing and increasing leptin values in the acute phase (within 14 days) in critically ill patients have previously been reported (Papathanassoglou et al. 2001; Grigoras et al. 2014; Yousef et al. 2010). However, only one previous study has separated the results between male and female patients, finding higher leptin values in septic male patients (Chen et al. 2014). The distribution between male and female subjects in a study cohort may influence the result.

Several studies have investigated and found an association between elevated leptin levels and unfavourable outcome in cerebral emergencies such as ICH, ischemic stroke and SAH (Kim et al. 2012; Dong et al. 2010; Fan et al. 2013; Zhao et al. 2012; Zhang et al. 2013). Increased leptin levels have been found to be an independent predictor for mortality in ICH (Zhao et al. 2012; Zhang et al. 2013). Elevated leptin levels in this study were not associated with unfavourable outcome defined as GOS 1–3, neither in female nor in male patients. However, leptin levels were significantly higher in the four female patients who deceased compared to female survivors. This association with higher leptin levels in deceased patients was not found in male patients. Previous studies looking at leptin in relation to outcome have not analysed female and male patients separately, although it is well known that leptin levels are higher in females from early childhood (Kim et al. 2012; Dong et al. 2010; Fan et al. 2013; Zhao et al. 2012; Zhang et al. 2013).

The difference found in this study, with diverse patterns of reaction depending on gender, is intriguing and not easily understood. Different patterns depending on gender have previously been reported in studies on stroke and diabetes, which may suggest that this is more than an accidental finding (Soderberg et al. 2004, 2007).

Experimental studies have shown that the brains of male and female rats are differently sensitive to the catabolic actions of small doses of leptin (and insulin), and estrogens can alter the hypothalamic sensitivity for leptin (Clegg et al. 2003; Meli et al. 2004). Whether or not this is relevant for the associations of leptin described in this study is to our knowledge unknown. In addition, leptin may be a significant factor for sex-related differences in the development of inflammation and dysfibrinolysis in the vessel wall (Lloyd-Jones et al. 1999).

The leptin production after administration of intravenous lipids is ambiguous and both decreased production of leptin and no change at all have been reported (Garcia-Lorda et al. 2003; Evans et al. 2001; Marana et al. 2008). In this study we investigated leptin before and after administration of lipid containing parenteral nutrition or lipid containing propofol and no effect on leptin levels were found before and after administration of lipids intravenously.

Even though leptin levels and regulation seem to be closely linked to both prediction and prognosis of cerebrovascular diseases such as stroke and SAH, knowledge about circulating levels of leptin has yet not found its clinical implication. However, our findings are in line with previous reports from our group suggesting that leptin does have an important role in vascular pathophysiology in the brain and further mechanistic research is needed to further describe this, not least the intriguing sex-related differences.

Finally, the results from this study only apply to SAH patients.

## Conclusion

Compared to controls, leptin levels were significantly higher in SAH patients at admission. Leptin levels were also significantly higher in female patients compared to male patients. Higher leptin levels were associated with increasing SOFA score, as a sign of increasing organ failure, in female but not in male patients. Female non-survivors also had significantly higher leptin levels compared to female survivors. Differences in leptin levels related to organ failure and death were not found in male patients. The known difference in leptin levels between men and women has to be considered when results from studies are presented.
